# Monitoring and influencing factors of grassland livestock overload in Xinjiang from 1982 to 2020

**DOI:** 10.3389/fpls.2024.1340566

**Published:** 2024-03-27

**Authors:** Lisha Ma, Jianghua Zheng, Jian Pen, Xianghua Xiao, Yujia Liu, Liang Liu, Wanqiang Han, Gangyong Li, Jianli Zhang

**Affiliations:** ^1^ College of Geography and Remote Sensing Science, Xinjiang University, Urumqi, China; ^2^ Key Laboratory of Oasis Ecology, Xinjiang University, Urumqi, China; ^3^ Xinjiang Uygur Autonomous Region Grassland Station, Urumqi, China

**Keywords:** aboveground biomass, theoretical livestock carrying capacity, grass-livestock balance, geographic detector, sustainable development

## Abstract

It is crucial to estimate the theoretical carrying capacity of grasslands in Xinjiang to attain a harmonious balance between grassland and livestock, thereby fostering sustainable development in the livestock industry. However, there has been a lack of quantitative assessments that consider long-term, multi-scale grass-livestock balance and its impacts in the region. This study utilized remote sensing and empirical models to assess the theoretical livestock carrying capacity of grasslands. The multi-scale spatiotemporal variations of the theoretical carrying capacity in Xinjiang from 1982 to 2020 were analyzed using the Sen and Mann-Kendall tests, as well as the Hurst index. The study also examined the county-level grass-livestock balance and inter-annual trends. Additionally, the study employed the geographic detector method to explore the influencing factors. The results showed that: (1) The overall theoretical livestock carrying capacity showed an upward trend from 1982 to 2020; The spatial distribution gradually decreased from north to south and from east to west. In seasonal scale from large to small is: growing season > summer > spring > autumn > winter; at the monthly scale, the strongest livestock carrying capacity is in July. The different grassland types from largest to smallest are: meadow > alpine subalpine meadow > plain steppe > desert steppe > alpine subalpine steppe. In the future, the theoretical livestock carrying capacity of grassland will decrease. (2) From 1988 to 2020, the average grass-livestock balance index in Xinjiang was 2.61%, showing an overall increase. At the county level, the number of overloaded counties showed an overall increasing trend, rising from 46 in 1988 to 58 in 2020. (3) Both single and interaction factors of geographic detectors showed that annual precipitation, altitude and soil organic matter were the main drivers of spatiotemporal dynamics of grassland load in Xinjiang. The results of this study can provide scientific guidance and decision-making basis for achieving coordinated and sustainable development of grassland resources and animal husbandry in the region.

## 1 Introduction

Grasslands play a crucial role as an essential component of the grassland ecosystem, serving as a primary source for livestock production and sustaining ecological balance. It not only provides food and habitat for animals but also serves important functions such as retaining soil moisture and preventing soil erosion (Yan et al., 2023). Ensuring the balance of grassland ecosystems and fostering sustainable development in the livestock industry are of paramount importance. Overgrazing and climate change, along with their combined effects, have been identified as the primary causes of approximately 49% of global grassland degradation and biodiversity decline (Wu et al., 2023b). Therefore, Performing precise evaluations on how livestock production and grassland productivity are interrelated, and understanding the spatiotemporal distribution of the balance between grassland and livestock, and exploring the factors influencing it can offer significant scientific groundwork for establishing sustainable livestock production and development (Song et al., 2022).

The concept of carrying capacity was introduced as a means to regulate grazing intensity and address the issue of overgrazing on grasslands. It refers to the “ecologically sustainable stocking rate that considers vegetation production, site ecology, and animal needs” (Qin et al., 2021). The carrying capacity of grassland ecosystems can be assessed by measuring indicators such as vegetation coverage, aboveground biomass, species diversity, soil quality, and water resource utilization (Zhang and Zhang, 2014). If the carrying capacity of a grassland ecosystem exceeds its load capacity, it can lead to issues such as grassland degradation, land desertification, and loss of biodiversity (Wei et al., 2022). Xinjiang, located in the heart of Central Asia, enjoys ample sunlight and abundant heat, which creates favorable conditions for the growth of grasslands and provides excellent opportunities for the development of livestock production (Xiuping et al., 2019). Certainly, there are challenges like sparse vegetation, poor soil quality, and the high sensitivity of grassland ecosystems to climate change and human activities in Xinjiang (Chen et al., 2019). Furthermore, due to escalating human demands and the impact of climate change, the extent of overgrazing has intensified, leading to substantial harm to grassland ecosystems. This has led to severe degradation of grasslands, loss of carbon, and environmental pollution (Wang et al., 2022b). Historically, studies on aboveground biomass and stocking capacity in grasslands were constrained by early data limitations, primarily focusing on short-term research and emphasizing vegetation growth conditions (Godde et al., 2020; Wolf et al., 2021; Wei et al., 2023); However, the evaluation of long-term series and multiple time scales can better reflect the past and future development trends of livestock carrying capacity.

Remote sensing technology is commonly used for the monitoring of vegetation, particularly in grassland ecosystems. By integrating field surveys in typical regions, it facilitates enduring and dynamic investigations on the vegetation biomass of grassland ecosystems spanning large to medium spatial scales (Wang et al., 2019). Ground biomass data for grasslands can be obtained through on-site measurements, but it becomes challenging to collect data at a regional scale, leading to significant limitations (Zhou et al, 2023). Existing regional-scale studies often rely on biomass and grassland productivity data to estimate theoretical carrying capacity using remote sensing methods (Zhou et al., 2023). Currently, the use of remote sensing models is a common method for estimating large-scale grassland productivity, which is then used to further estimate the maximum sustainable population size supported by the grassland. The estimation of regional aboveground biomass is primarily based on NDVI (Hunt and Miyake, 2006; Yu et al., 2010; Wang et al., 2022a) and NPP (Net Primary Productivity) data (Qian et al., 2012; de Leeuw et al., 2019; Piipponen et al., 2022). Prior research has demonstrated that NPP is a more direct productivity indicator, taking into account plant growth, respiration and nutrient utilization; For calculating theoretical carrying capacity, NPP can be utilized to assess the potential food availability for animals (aboveground biomass), thereby assisting in determining the number of livestock that grasslands can support. The aboveground biomass in grasslands serves as the primary food source for grazing livestock. It is a material foundation for the development of grazing livestock and an important measure of evaluation of carrying capacity. It facilitates the calculation of the ideal livestock population that can be grazed within a given timeframe (Xianzhou et al., 2022). However, in a long time series and across multiple scales, the quantitative assessment of the grass-livestock balance situation and its influencing factors in Xinjiang has not yet been conducted. Additionally, Given the substantial spatial and temporal variations in regional grasslands, models simulating grassland carrying capacity at the regional level inherently encounter limitations and uncertainties (Jia et al., 2016).

The carrying capacity of grassland ecosystems is impacted by a range of factors, including climate conditions, soil quality, water resources, vegetation types, and human activities. Currently, there is insufficient analysis of the spatiotemporal driving factors for grassland carrying capacity, and there is a lack of qualitative or quantitative research on the single or interactive effects of spatiotemporal dynamics on carrying capacity. Furthermore, most existing studies predominantly analyze the impact of climate factors on grassland carrying capacity while overlooking the socio-economic and human activity factors such as topography, livestock density, population GDP, and more (Zeng et al., 2021; Ding et al., 2022; Wang, 2022). Therefore, quantitatively investigating the single or interactive effects of factors on the carrying capacity of the grassland ecosystem in Xinjiang is a crucial task for protecting the region’s ecological environment and achieving sustainable development.

Sustainable Development Goal 15 (SDG 15) aims at the protection, restoration and sustainable management of land ecosystems, the promotion of sustainable forestry practices, the fight against desertification, the cessation and reversal of soil degradation, and the cessation of biodiversity loss. It is crucial to attain these objectives to ensure the sustainability of the environment and the health of our planet (Zhang et al., 2023b). The challenges posed by deforestation and desertification, resulting from human activities and climate change, are indeed substantial obstacles to achieving the Sustainable Development Goals. These challenges have consequences not only for the environment but also for the well-being of millions of individuals and initiatives aimed at reducing poverty. In order to respond to and support the achievement of this goal in a timely manner and meet the needs of sustainable development, this study focuses on revealing the increasing trend and causes of overgrazing in grasslands in Xinjiang. It aims to explore the estimation of the theoretical carrying capacity of grasslands in Xinjiang, which is crucial for understanding the grass-livestock balance in arid and semi-arid regions.

Based on the above, this study focuses on investigating the current situation of overgrazing in Xinjiang grasslands from 1982 to 2020, as well as the influencing factors. The aim is to explore the following three aspects: (1) Evaluate the spatial distribution and future trends of monthly, seasonal, annual, and different grassland types’ theoretical carrying capacity in Xinjiang from 1982 to 2020. (2) Investigate the current grass-livestock balance in Xinjiang from a county-level perspective and examine the interannual variations by incorporating data from statistical yearbooks. (3) Quantitatively explore the impact of climate change and human activities, as well as socio-economic factors, on the spatiotemporal dynamics of the theoretical carrying capacity of Xinjiang grasslands.

## 2 Materials and methods

### 2.1 Study area

**Figure 1:**
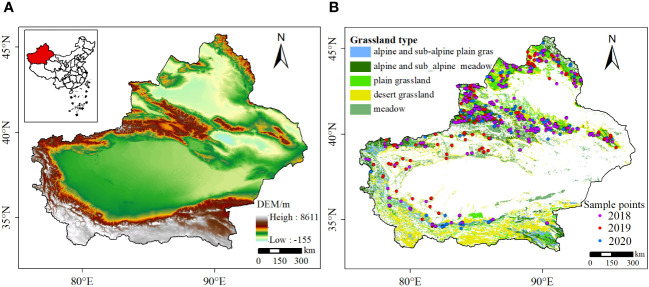
Study area. **(A)** DEM. **(B)** Vegetation types in Xinjiang from 1982 to 2020.

Xinjiang is located in the northwest region of China, with geographical coordinates ranging from 73°40′ to 96°18′E and 34°25′ to 48°10′N. It covers a total area of approximately 1.6649 million square kilometers. The region is characterized by alternating mountain ranges and basins, forming a unique “three mountains, two basins” topography (**Figure 1A**). Xinjiang is located in an inland region and is surrounded by high mountains, which are far away from the ocean. In Xinjiang, the northern part is dominated by the Altai Mountains, the southern part is characterized by the Kunlun Mountain range, and the central part is the Tianshan Mountains. These mountain ranges divide Xinjiang into the Tarim Basin in the south and the Junggar Basin in the north (Han et al., 2023). The geographical environment in Xinjiang gives rise to a typical temperate continental climate, where maritime air masses have limited reach. As a result, Xinjiang experiences large temperature variations, with hot and dry summers and cold and dry winters. The region also has long hours of sunshine annually, relatively low precipitation, and high evaporation rates (Duan et al., 2017). The annual average temperature in Xinjiang ranges from 9 to 12°C, with an average annual precipitation of approximately 100-200 millimeters (Liu et al., 2023b). Due to its diverse and complex topography, Xinjiang is rich in grassland resources. The grassland types in Xinjiang mainly include alpine meadows, alpine grasslands, desert grasslands, steppe grasslands, and meadows (Zhang et al., 2023a) (**Figure 1B**). Overall, Xinjiang is considered a typical arid and semi-arid region with low vegetation coverage in its ecosystems. This makes it highly sensitive to climate change and human activities. Changes in climate patterns and human interventions can have significant impacts on the fragile ecological balance in Xinjiang (He et al., 2021).

### 2.2 Data collection and preprocessing

#### 2.2.1 NDVI datasets

For this study, the selected NDVI dataset is the NASA MOD13A3 dataset (https://ladsweb.modaps.eosdis.nasa.gov/search/) covering the period from 2000 to 2020. The data has a monthly temporal resolution and a spatial resolution of 1 km. Additionally, the study also utilizes the GIMMS NDVI 3g dataset (https://ecocast.arc.nasa.gov/data/pub/gimms/) released by the NASA Global Inventory Monitoring and Modeling System Group. The data has a temporal resolution of 15 days and a spatial resolution of 1/12°, spanning from 1982 to 2015.

#### 2.2.2 Climate datasets

The primary source of climate data utilized in this research is the National Science & Technology Infrastructure Platform of China’s National Earth System Science Data Center (http://www.geodata.cn). The dataset comprises data for monthly average temperature and precipitation for China at 0.01°, covering the period 1901-2021 with a resolution of 1 km.

The solar radiation data for the period from 1982 to 2020 was obtained using the Famine Land Data Assimilation System (FLDAS) dataset (https://ldas.gsfc.nasa.gov/index.php/fldas/). The Palmer Drought Severity Index (scPDSI), which is derived from the University of East Anglia drought data set in the United Kingdom, has been developed(https://crudata.uea.ac.uk/cru/data/drought/). It has a spatial resolution of 0.5° and a temporal resolution of one month. The data has been re-sampled to a 1 km spatial resolution using the resampling technique. The scPDSI is a widely used index for assessing drought severity and monitoring drought conditions based on a balance between moisture supply and demand.

#### 2.2.3 Vegetation type and topographic data

The land cover data used in this study is sourced from the Global Land Cover 2000 (GLC2000) product developed by the Joint Research Centre (JRC) of the European Union(https://forobs.jrc.ec.europa.eu/). The dataset providing detailed information on land cover characteristics. In this study, the land use types in Xinjiang have been reclassified, resulting in a grassland type map that encompasses five main categories: alpine and subalpine meadows, steppe grassland, desert grassland, meadow, and alpine and subalpine grassland. The Digital Elevation Model (DEM) is a component of the National Science & Technology Infrastructure of China, which is the National Earth System Science Data Center. The dataset can be accessed at (http://www.geodata.cn) and is a valuable resource for obtaining elevation information for the study area. Specifically, the data utilized is the Chinese 30-meter DEM (ASTER DEMv3). The DEM data is used to calculate the slope and aspect of the study area, with a spatial resolution of 30 meters. The soil organic matter data (http://globalchange.bnu.edu.cn/research/soil2) is derived from the Chinese Soil Characteristics Dataset, which was released by the Research Group on Land-Atmosphere Interactions at Sun Yat-sen University.

#### 2.2.4 Others datasets

The population density data is obtained from two sources: the Resource and Environmental Science and Data Center of the Institute of Geographic Sciences and Natural Resources Research, Chinese Academy of Sciences (1990-2010), and WorldPop (2000-2020) (https://hub.worldpop.org/project/categories?id=18). The GDP data is selected from the China GDP Spatial Distribution Kilometer Grid Dataset (1995-2019) (https://www.resdc.cn/data.aspx?DATAID=252). The actual carrying capacity data is sourced from the “Statistical Yearbook of Xinjiang” and the “Livestock Inventory at the end of each year in various regions, cities, counties (cities), and troops” in the statistical yearbooks of various cities and counties. Since the data in the statistical yearbooks only starts from 1988 and is reported at the county level, the assessment of the grass-livestock balance in this study can only be conducted for the period of 1988-2020.

Based on the inconsistent spatial resolutions of all the datasets, the bilinear interpolation method, which is a resampling technique, was used in this study to standardize all the datasets to a spatial resolution of 1km. This was done to ensure consistency in spatial resolution for subsequent processing and computation.

### 2.3 Methods

#### 3.3.1 CASA model

The paper utilizes the Carnegie-Ames-Stanford Approach (CASA) to estimate NPP. The CASA model, which is a light use efficiency model, integrates multiple factors such as NDVI, solar radiation, temperature, precipitation, and vegetation type. This comprehensive approach enables a more accurate assessment of NPP. The formula is as follows (Liu et al., 2023b):


(1) $$NP{\text{P}}_{\left(\text{x},\text{t}\right)}=APA{\text{R}}_{\left(\text{x},\text{t}\right)}+{\text{ε}}_{\left(\text{x},\text{t}\right)}$$

In the Equation (1), NPP(x, t) indicates the Net Primary Productivity at position x in month t. APAR(x, t) denotes the absorbed photosynthetically active radiation (MJ/m^2^), and (x, t) represents the actual light use efficiency (gC/MJ).

#### 2.3.2 Time series interpolation

To ensure the continuity of NDVI data, the overlapping period data from the GIMMS NDVI and MOD13A3 time series, specifically the 2000-2015 NDVI data, can be used. By constructing a linear regression model between the two datasets, the NDVI data can be effectively interpolated, maintaining its seamless nature (Guan et al., 2021). The interpolation of time series is performed using the following Equation (2):


(2) $$G_{i}=\text{a}+bV_{i}+\varepsilon_{i}$$

The random error *ε_i_*, model parameters a and b are utilized in the time series interpolation. The variables are computed using the least squares method, obtaining the optimal regression equation through the following expression (Liu et al., 2023a):


(3) $$b=\frac{\sum_{i=1}^{n}\left(G_{i}-\bar{\text{V}}\right)\left(G_{I}-\bar{\text{G}}\right)}{\sum_{i=1}^{n}{\left(V_{i}-\bar{\text{V}}\right)}^{2}}$$

(4) $$\text{a}=\bar{\text{G}}-\text{b}\bar{\text{V}}$$

In the provided Equations (3, 4), $G_{i}$ represents the resampled GIMMS NDVI for the i-th month, $V_{i}$ represents the MOD13A3 NDVI for the i-th month, and G and V are the mean values of GIMMS and MODIS NDVI, respectively, for all months from 2000 to 2015, at a pixel scale.

#### 2.3.3 The calculation of theoretical carrying capacity for livestock

In order to convert biomass into carbon, the coefficient of 0.47 is used to convert NPP values into grassland yield. The following formula is used to derive the allocation of NPP to AGB (fANPP) (Hui and Jackson, 2006) as shown in Equation (5):


(5) $$F_{i}=\frac{NPP\times fANPP}{0.47}$$

fANPP=0.171+0.0129MAT

Where Fi represents the grassland yield in grams per square meter (g/m^2^), and MAT represents the monthly average temperature in degrees Celsius (°C).

Before calculating the theoretical carrying capacity, it is necessary to convert the unit of AGB to kg/hm^2^ (Piipponen et al., 2022), 1g/m^2^ = 10kg/hm^2^ as in Equation (6).


(6) $$CC=\frac{AGB\times G_{i}}{\text{L}\times \text{D}}$$

CC represents the theoretical carrying capacity, AU/hm^2^; AGB refers to the yield of dry forage per unit area, kg/hm^2^; Gi represents the utilization rate of the i-th type of grassland; L represents the standard daily forage intake per sheep, 1.8 kg/d; D represents the number of days the grazing is done.

#### 2.3.4 Forage-livestock balance index

The actual carrying capacity is determined based on the livestock population at the end of the year in each county or city of the region, as recorded in the statistical yearbook. The livestock population is then converted to sheep units, using the following conversion factors: 1 head of cattle equals 5 sheep units, 1 horse equals 5 sheep units, 1 donkey equals 2.5 sheep units, 1 goat equals 0.8 sheep units, 1 sheep equals 1.2 sheep units, 1 camel equals 8 sheep units, and 1 mule equals 5 sheep units (Pandey and Upadhyay, 2022).


(7) $$I_{P}=\frac{A-CA}{CA}\times 100\%$$

In the Equation 7, I_P_ represents the Forage-Livestock Balance Index, A represents the actual carrying capacity, and CA represents the theoretical carrying capacity.

The classification standards for the state of forage-livestock balance are formulated based on the national standard GB51 T1480-2012, combined with the grazing characteristics of grasslands in Xinjiang (**Table 1**).

**Table 1:** Classification standards for forage-livestock balance status.

Grade code	Overload rate	Grade
I	I_p_<0%	Not overloaded
II	0%<I_p_≤25%	Slight overload
III	25%<I_p_≤50%	Moderate overload
IV	50%<I_p_	Severe overload

#### 2.3.5 Sen’s trend and Mann-Kendall test

The Theil-Sen Median method, also called Sen’s Slope Nonparametric Method, is a highly efficient method with low error and outliers. Therefore, it is commonly employed for trend analysis in long time series data (Agarwal et al., 2021). The Mann-Kendall method is a powerful non-parametric statistical test that does not rely on normal distribution or linear trend assumptions (Li et al., 2023b).

#### 2.3.6 Geographical detector

The geographical detector is a statistical instrument used for evaluating spatial variations and determining the factors that influence them. These detectors enable the measurement and exploration of spatial variations and their underlying determinants in a comprehensive manner (Ge et al., 2022). The factor detector is mainly utilized for assessing the influence of a factor (X) on the explained factor (Y). Conversely, the interaction detector is employed to identify the combined impact of two factors (X) on the outcome (Y) (Li et al., 2023c). This article employs factor and interaction detectors to assess the impacts of natural and human activities on the carrying capacity of grassland for livestock. Additionally, they help identify whether the interaction between any two factors amplifies or diminishes their effects on the carrying capacity. Due to limitations in socio-economic data, the analysis of the driving factors determining the annual variations in theoretical carrying capacity of grassland will be conducted using data from specific time points. The selected time points for analysis will be 1990, 2000, 2010, and 2019. In the analysis of driving factors, seven natural factors will be considered: average annual temperature (X1), annual precipitation (X2), Standardized Precipitation Evapotranspiration Index (scpdsi) (X3), altitude (X4), slope (X5), aspect (X6), and soil type (X9). Additionally, two socio-economic factors, population density (X7) and Gross Domestic Product (GDP) (X8), will also be included in the analysis.

## 3 Results

### 3.1 Simulation NPP accuracy verification

**Figure 2:**
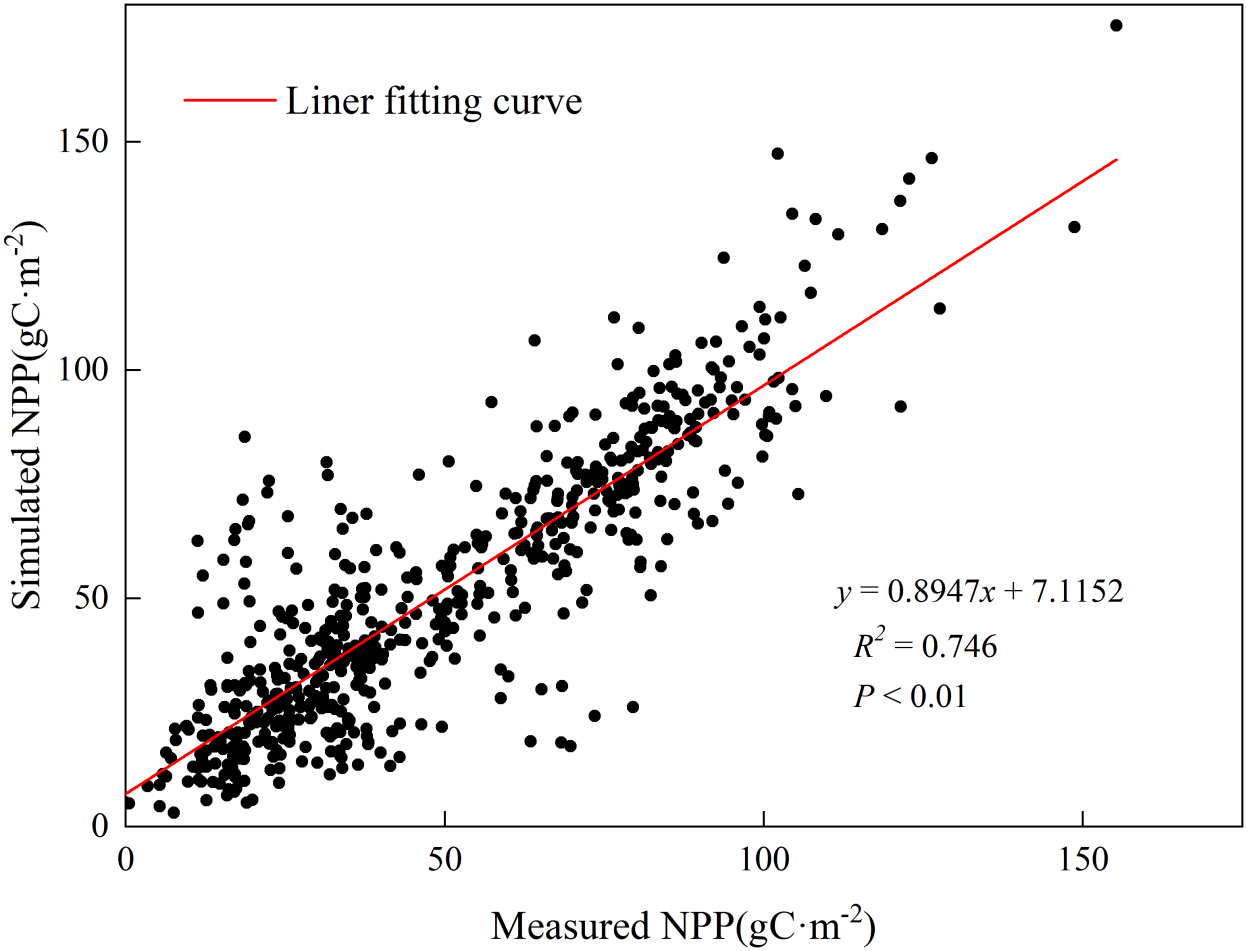
NPP model accuracy validation.

The measured biomass data for the entire Xinjiang region were provided by the Xinjiang Grassland Station from 2018 to 2020. The collection of aboveground biomass field measurements was conducted by setting up 1m×1m quadrats in various locations across Xinjiang during the months of June and July each year from 2018 to 2020. A sum of 617 valid sample points was acquired. The spatial distribution of these sample points is shown in **Figure 1B**, with 200 sample points in 2018, 222 sample points in 2019, and 195 sample points in 2020. After comparing the simulated Net Primary Productivity (NPP) from the CASA model with the measured NPP data from 2018 to 2020, it was found that there is a significant correlation (R^2^ = 0.746, *P*<0.01) between the simulated NPP results and the measured data, as indicated by the fitting results shown in **Figure 2**. The findings indicate that the NPP simulated using the CASA model can effectively reflect the grassland conditions in the study area. The estimation results demonstrate a high level of accuracy.

### 3.2 The spatial distribution of theoretical carrying capacity

#### 3.2.1 Month-scale spatial distribution

**Figure 3:**
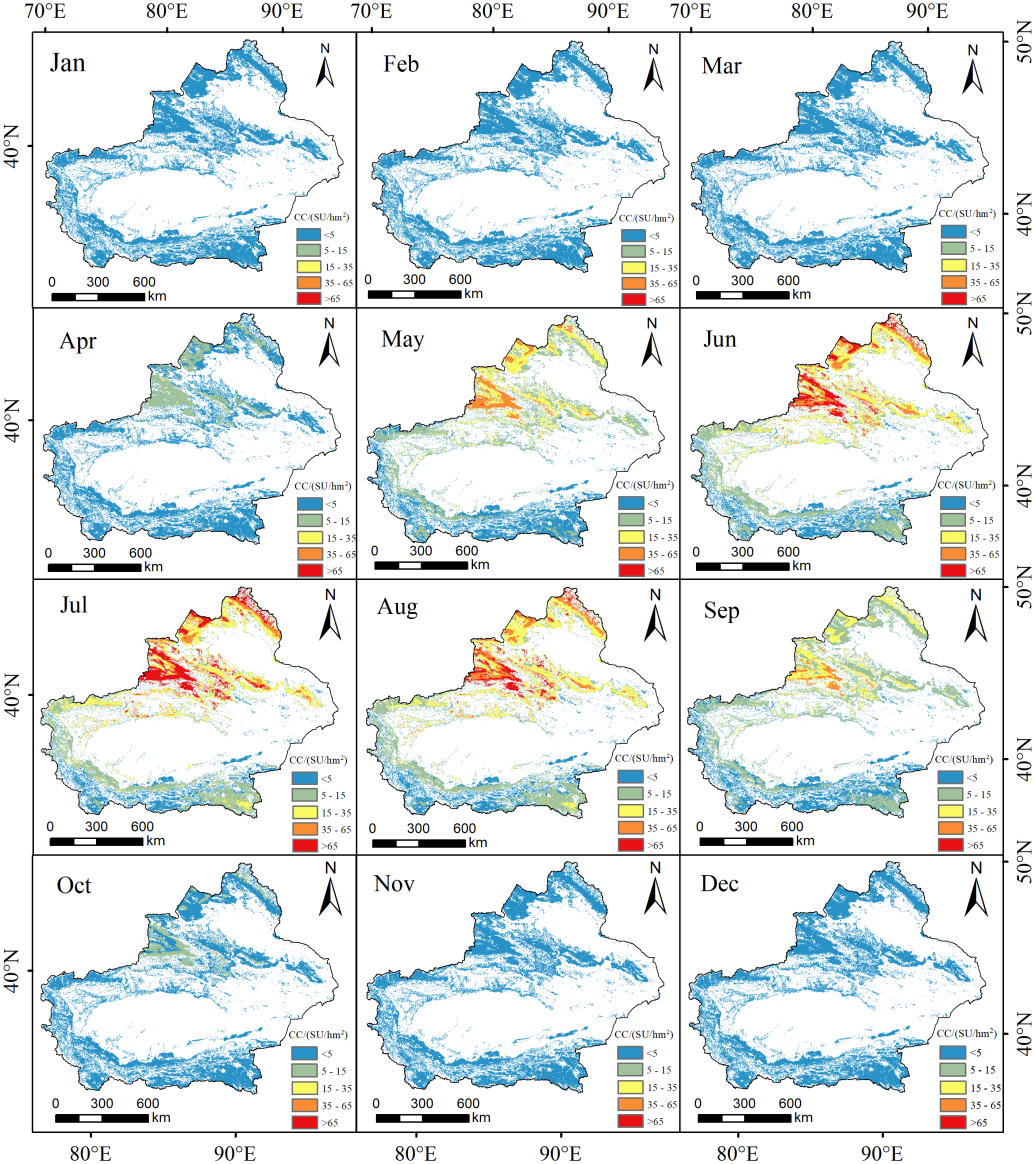
Spatial distribution of the mean monthly theoretical livestock carrying capacity from 1982 to 2020.

**Figure 3** displays the spatial distribution of the average theoretical carrying capacity for each month from 1982 to 2020. According to the temporal analysis, the theoretical carrying capacity exhibits low values (<5SU/hm^2^) during the months of January to March, November, and December, primarily due to the onset of the cold season and limited grassland growth. The carrying capacity notably increases from April, reaching its peak in July with an average of 25.59SU/hm^2^. Subsequently, there is a gradual decrease in the carrying capacity. Spatially, the theoretical carrying capacity demonstrates a gradually increasing trend from south to north, with the northern regions such as Yili and Altay in Xinjiang being the main high-value areas. This indicates that the grassland exhibits good growth status and quality in the mentioned areas. The favorable water and thermal conditions contribute to a stronger carrying capacity for livestock; The low-value areas are mainly distributed in most parts of southern Xinjiang, indicating a weaker carrying capacity for livestock in these regions.

#### 3.2.2 Seasonal-scale spatial distribution

**Figure 4:**
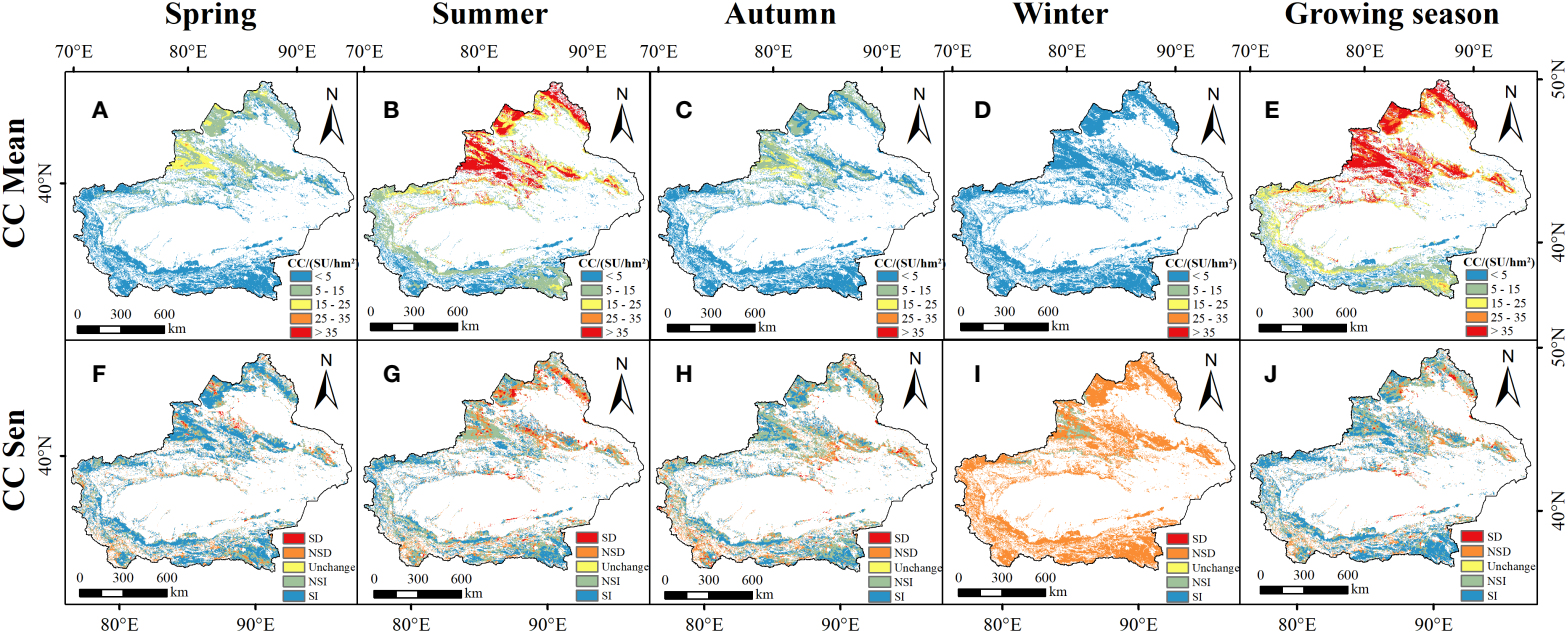
Spatial distribution of growing season **(E)** and seasonal **(A–D)** theoretical livestock carrying capacity means, Sen’trend and MK tests **(F–J)**. SD, significant decreased; NSD, non significant decreased; NSI, non significant increased; SI, significant increased.

According to **Figure 4**, it can be observed that the theoretical carrying capacity follows the order from highest to lowest: growing season>summer>spring>autumn>winter. The carrying capacity decreases during winter, while it increases during the other seasons. Throughout the period of plant growth, the theoretical carrying capacity ranges from 0 to 191 SU/hm^2^, with an average of 30 SU/hm^2^. The highest values are predominantly dispersed in the northern part of the Tianshan Mountains and high mountainous regions, where there is abundant rainfall. This provides optimal conditions for vegetation growth and development (**Figure 4E**), resulting in a higher carrying capacity for livestock. The lowest values are situated in the adjacent regions of the Taklamakan Desert. In spring, the theoretical carrying capacity ranges from 0 to 37 SU/hm^2^, with an average of 5 SU/hm^2^ (**Figure 4A**). As temperatures rise and precipitation increases, vegetation gradually begins to grow, leading to an improvement in the condition of grasslands. Consequently, the carrying capacity of grasslands for livestock tends to increase. Summer temperatures rise and ample sunlight is conducive to photosynthesis and growth of grassland plants, providing abundant forage supply for livestock and herbivores. Therefore, the theoretical carrying capacity for livestock in summer is typically higher than in other seasons, with an average of 21AU/hm^2^ (**Figure 4B**). In autumn, vegetation leaves begin to wither or wilt, resulting in a decrease in forage availability. As a result, the average carrying capacity for livestock in autumn is reduced to around 4AU/hm^2^ (**Figure 4D**). During winter, low temperatures and shorter daylight hours cause most vegetation to enter a dormant state and cease growth. As a result, the theoretical carrying capacity for livestock reaches its lowest point or may even be nonexistent during this season (**Figure 4D**).

The utilization of Sen’s trend analysis in conjunction with the MK test can offer a more precise evaluation of the change trend in Carrying Capacity (CC). The results indicate that the theoretical carrying capacity (CC) in all seasons of Xinjiang has predominantly increased from 1982 to 2020, with an increase percentage of over 70% in each season. This suggests that the grassland quality in various regions of Xinjiang has been improving year by year, consequently enhancing the livestock carrying capacity. The percentage increase in theoretical carrying capacity (CC) during spring and the growing season from 1982 to 2020 was 82.69% and 79.45% respectively (**Figures 4F, J**). All regions in Xinjiang exhibited a significant increasing trend in theoretical carrying capacity (CC), except for a few areas such as Hami, the southeast part of Altay, and Manas County, which experienced a slight significant decrease (17.31%). The percentage of the increasing trend in theoretical carrying capacity (CC) during the summer season is the lowest compared to other seasons (70.58%). The areas with a decreasing trend are mainly distributed in Hetian County, Weili County, and near the Altai Mountains (**Figure 4G**). The theoretical carrying capacity (CC) during the autumn season showed an increasing trend of 75.08%. The areas with a decreasing trend are mainly in the eastern periphery of the Tianshan Mountains, Hami, and near the Kunlun Mountains (**Figure 4H**). During the winter season, the theoretical carrying capacity (CC) showed a concentrated decrease in certain areas, primarily in the vicinity of the Yili River Valley and the Tachen region (**Figure 4I**).

#### 3.2.3 Year-scale spatial distribution of theoretical carrying capacity

**Figure 5:**
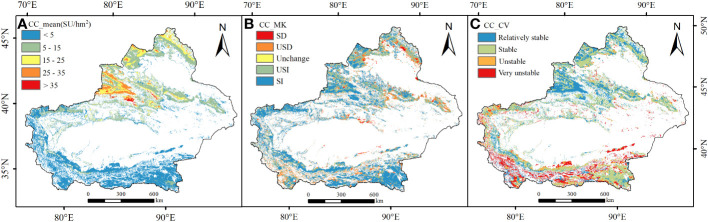
Spatial distribution of mean livestock carrying capacity **(A)**, Sen trend **(B)** and CV **(C)** during 1982 to 2020.

The theoretical carrying capacity (CC) depicted in **Figure 5** exhibits a gradual decline in both the north-south and east-west directions. The distribution range is from 0 to 47.70 SU/hm^2^, with an average value of 7.48 SU/hm^2^. The regions with higher carrying capacity are predominantly situated in proximity to mountainous areas of Yili, Tacheng, and Altay in Northern Xinjiang. The low-value areas are mostly found in the southern regions of Xinjiang (**Figure 5A**). The overall interannual trend is primarily characterized by an increase, accounting for 80.12% of the total distribution across the entire Xinjiang region. However, there is a decreasing trend representing 19.88% of the distribution, mainly observed in areas such as Yuli County, Qiemo County, Hami, and Qinghe County (**Figure 5B**). The spatial statistical analysis indicates that the coefficient of variation for the theoretical carrying capacity of grasslands in Xinjiang ranges from 0 to 5.11, with an average value of 0.18. This suggests that the theoretical carrying capacity of grasslands in Xinjiang is generally tending towards stability (**Figure 5C**). The proportion of areas with different levels of variation in descending order is as follows: stable (40.50%) > relatively stable (34.43%) > highly unstable (14.37%) > unstable (10.70%). This indicates that the theoretical carrying capacity of grasslands in Xinjiang exhibits spatial heterogeneity, with some regions experiencing higher levels of variation. These regions are primarily located near the mountainous areas in southern Xinjiang.

#### 3.2.4 Statistics of different grassland types

**Figure 6:**
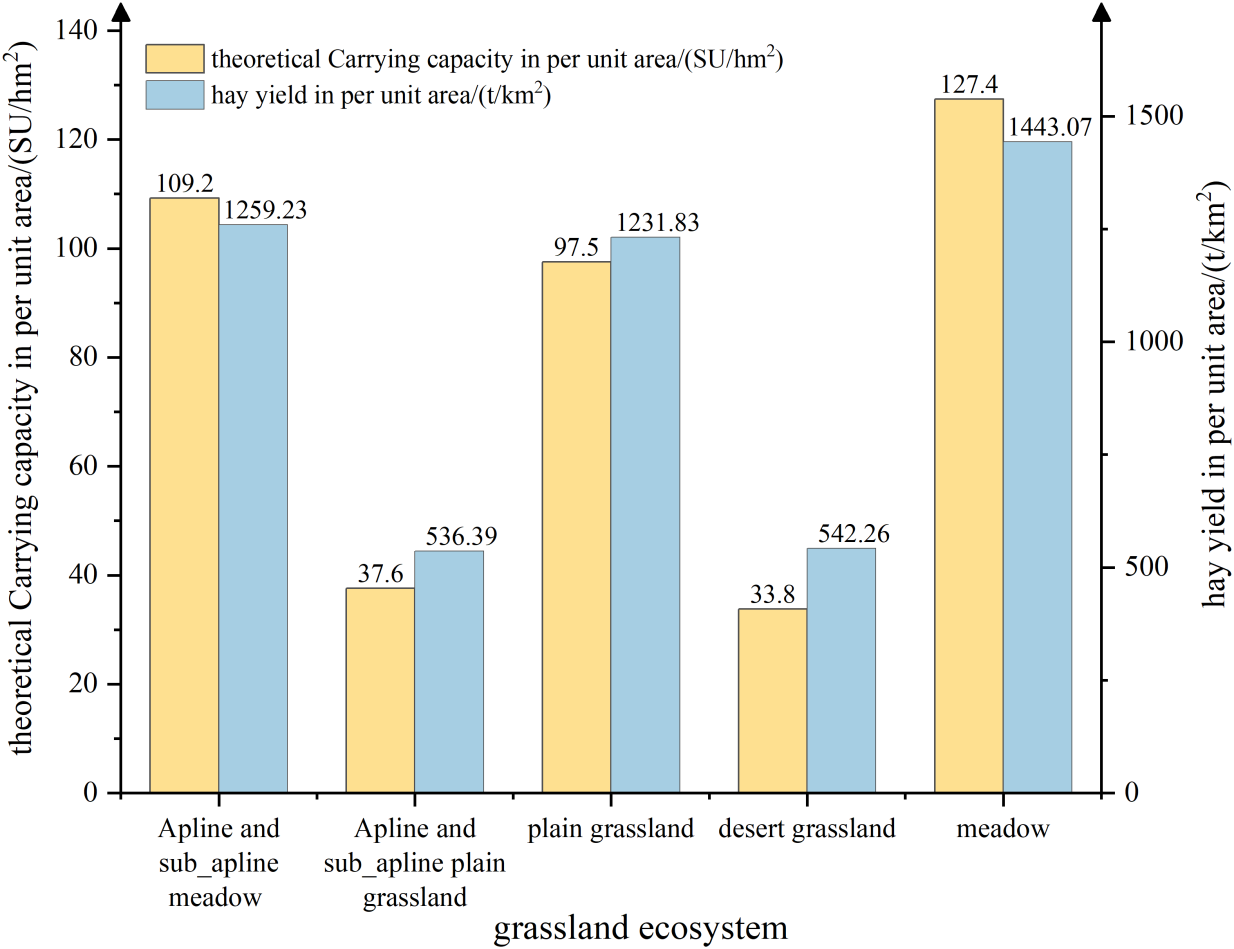
Total theoretical carrying capacity and hay yields of different grassland types during 1982-2020.

Based on the mathematical statistical analysis of the simulated results for forage production and theoretical carrying capacity (**Figure 6**), it has been determined that the average dry hay yield in Xinjiang is 901.88 t/km², and the average theoretical carrying capacity per unit area is 7.48 SU/hm². The five main types of grassland that contribute significantly to forage production are alpine and subalpine meadows, alpine and subalpine steppe, desert steppe, plain grassland, and meadow grassland. The annual dry hay yield per unit area, from highest to lowest, is as follows: meadow grassland > alpine and subalpine meadows > plain grassland > desert grassland > alpine and subalpine grassland. The annual theoretical carrying capacity per unit area, from highest to lowest, is as follows: meadow grassland > alpine and subalpine meadows > plain grassland > desert grassland > alpine and subalpine grassland. The grassland with the highest forage production and strongest carrying capacity is meadow grassland, with a production of 1443.07 t/km² and a theoretical carrying capacity of 127.4 SU/hm². Following that, alpine and subalpine meadows and plain grassland have similar forage production, with values of 1231.83 t/km² and 1259.23 t/km², respectively. The combined forage production of the top three grassland types, including meadow grassland, alpine and subalpine meadows, and plain grassland, accounts for 78.48% of the total forage production in Xinjiang. Similarly, these grassland types also have the highest carrying capacity, accounting for 81.57% of the total carrying capacity of all grasslands in the region.

### 3.3 Grass-livestock balance analysis

#### 3.3.1 Interannual trends

**Figure 7:**
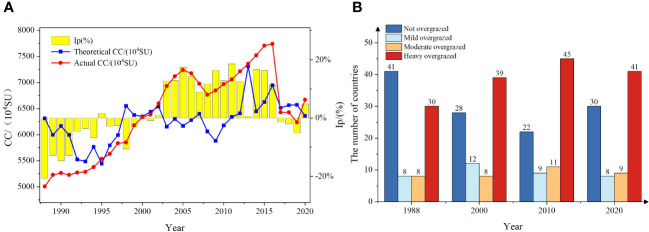
Liner trend of IP in Xinjiang from 1988 to 2020 **(A)** and country summary **(B)**.

From the temporal distribution (**Figure 7A**), it can be observed that the theoretical livestock load has fluctuated significantly over the past 33 years. It remained relatively stable during the period of 2017-2020 and saw the highest increase during 1995-1998 (11.02 million sheep units). On the other hand, the actual livestock load showed a continuous increasing trend from 1988 to 2016 and experienced a significant decrease from 2016 to 2017 (13.10 million sheep units). Over the past 33 years, the average grass-livestock balance index in Xinjiang has been 2.61%. There has been significant interannual variability, but overall, it has shown an upward trend, indicating a mild overloading state. This suggests that the grassland resources are gradually unable to meet the demands of livestock. In other words, the production capacity of the grassland is lower than the consumption capacity of the livestock. This indicates a shortage of forage production and an insufficient supply of feed for the livestock. The grass-livestock balance index can be broadly categorized into three stages: from 1988 to 2001, it was in an underloading state; from 2001 to 2016, it remained in a mild overloading state; and from 2016 onwards, there has been an improving trend. In 1988, the grass-livestock balance index reached its lowest point with a value of -20.70% (not overloaded). In 2020, the index value was 4.87% (mildly overloaded). During this period, the highest point was reached in 2011 with a balance index of 18.03% (mildly overloaded). From 1988 to 2016, the grass-livestock balance index showed a gradual upward trend with fluctuations. The theoretical livestock load also exhibited a fluctuating upward trend during this period. This indicates that the grassland condition was relatively stable. However, the actual livestock load exceeded the threshold of the theoretical livestock load. From 2016 to 2020, the grass-livestock balance index exhibited a downward trend, and the theoretical livestock load also decreased. This indicates a reduction in the actual livestock load on the grassland compared to the previous period. However, overall, the grassland still remained in a state of mild overloading.

#### 3.3.2 Spatial changes at the county level

**Figure 8:**
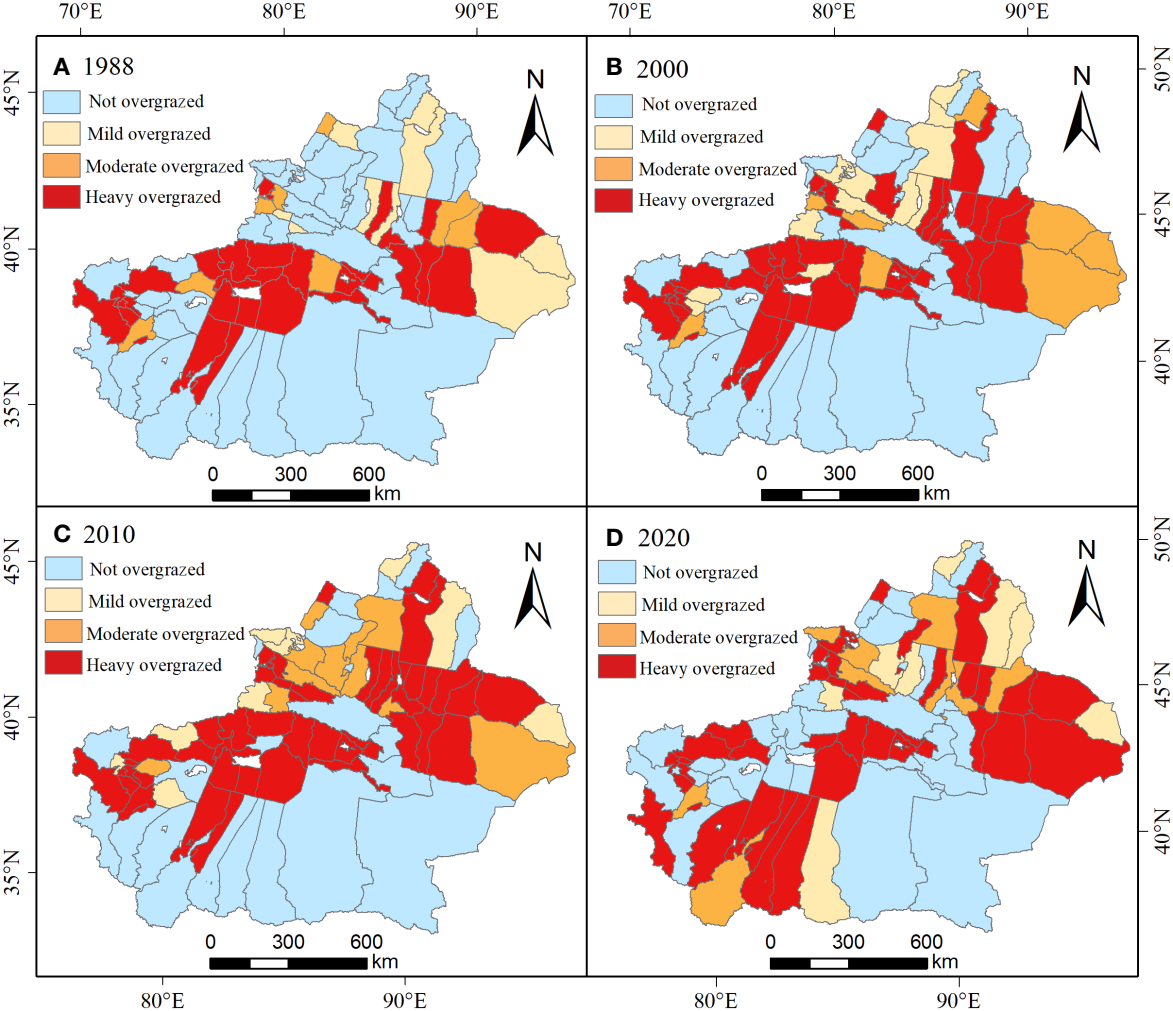
The spatial distribution and change of IP from 1988 to 2020, **(A–D)** correspond to the years 1988, 2000, 2010, and 2020 respectively.

According to the analysis of the grass-livestock balance index at the spatial county level (**Figures 7B**, **8**), it was found that during the period from 1988 to 2020, there were 17 counties that consistently remained in a state of severe overloading. These counties include Xinhe County, Moyu, Luopu, Shule, Shufu, Yingjisha, and Zepu County (**Figure 8**). In 1988, there were 46 counties (52.87%) that were in a state of overloading, while 41 counties were not overloaded. The counties that were primarily not overloaded were mainly distributed in Qiemo, Ruoqiang, and Tacheng areas (**Figure 8A**). Compared to 1988, the number of overloaded counties increased from 46 to 59 and 65 in 2000 and 2010 respectively, showing a gradual upward trend. The overloaded areas are mainly distributed in the Ili and Tacheng regions. For example, Wusu County changed from mild overload to moderate overload and then to severe overload. Yizhou District, Fuhai County, and other areas also transitioned from mild overload to moderate and severe overload (**Figures 8B, C**). In contrast, the number of overloaded counties in 2020 showed a slight decrease, dropping from 65 in the previous two years to 58 (**Figure 7B**). The areas that showed significant improvement were Aksu and Tacheng regions. However, regions such as Hotan experienced varying degrees of worsening overload conditions (**Figure 8D**).

### 3.4 Driving factors

#### 3.4.1 Factor detector

**Figure 9:**
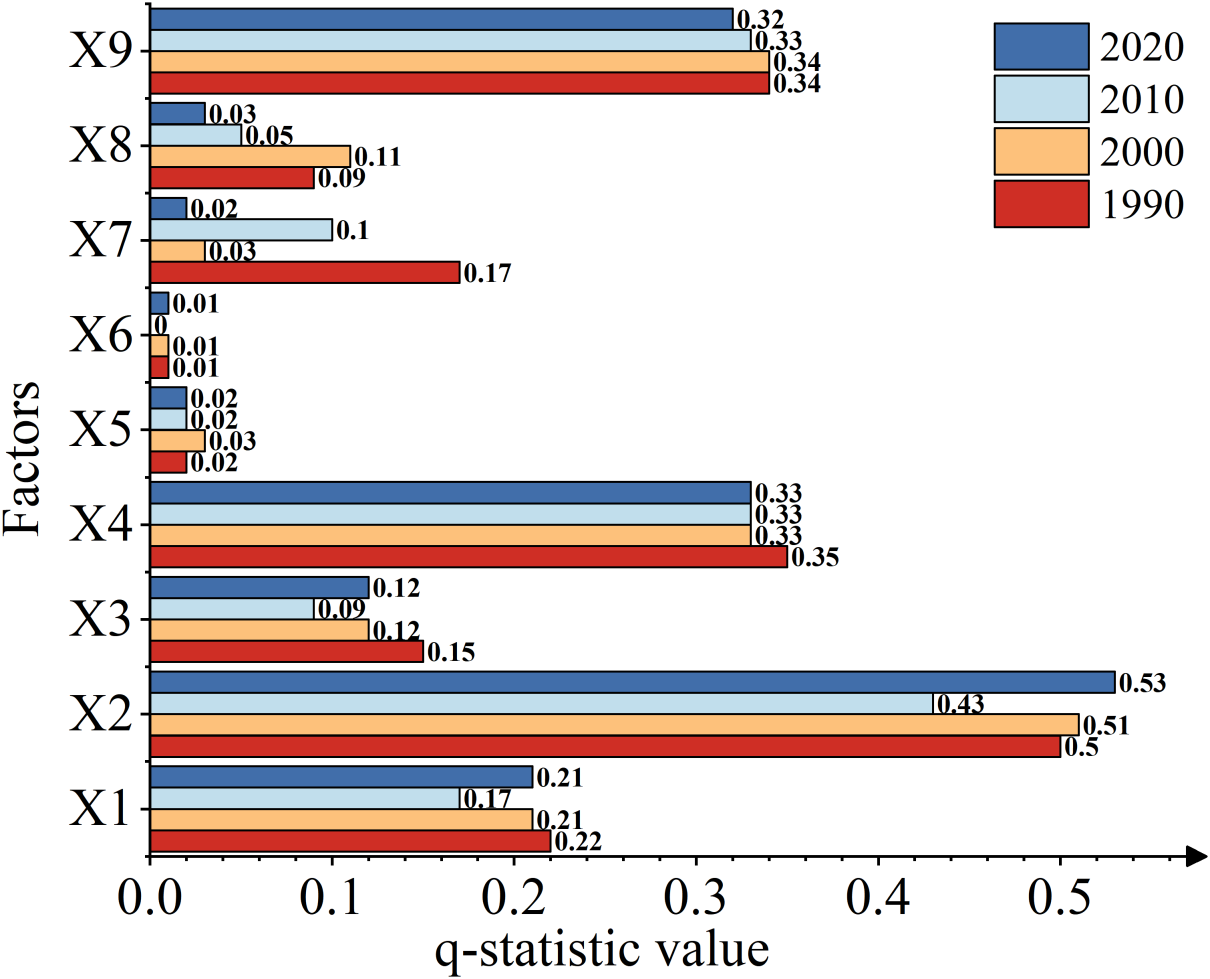
The q statistic values of driving factors in 1990,2000,2010 and 2020 in Xinjiang. All q statistic values met the conditions for P<0.01(X1-X9: Theoretical livestock carrying compacity, Average temperature, Precipitation, sc_pdsi, Elevation, Slope, Aspect, Population density, GDP, SOM).

Geographical Detector Single-factor measures the driving impact of various indicators on grassland carrying capacity changes by calculating the influence q-values representing their influence. The detection results show that the nine driving factors, ranked in descending order of q-values in 1988, 2000, 2010, and 2019, are as follows: annual precipitation > elevation > soil organic matter > annual average temperature > ScPDSI > population density > GDP > slope > aspect (**Figure 9**). Among them, annual precipitation and elevation have relatively large q-values, reaching 0.49 and 0.34 respectively in the four-year average. This indicates that they have a significant impact on grass production and indirectly affect grassland carrying capacity. Soil organic matter has a relatively lesser impact on grassland, with a three-year average value of 0.33. The influence of annual average temperature and ScPDSI on theoretical grassland carrying capacity is relatively small, with four-year average q-values of 0.20 and 0.12 respectively. Indeed, population density, GDP, slope, and aspect have the least impact on the theoretical grassland carrying capacity, with four-year average q-values all below 0.10. Therefore, annual precipitation, elevation, and soil organic matter are the main determining factors that influence the dynamics of theoretical grassland carrying capacity.

#### 3.4.2 Interaction detector

**Figure 10:**
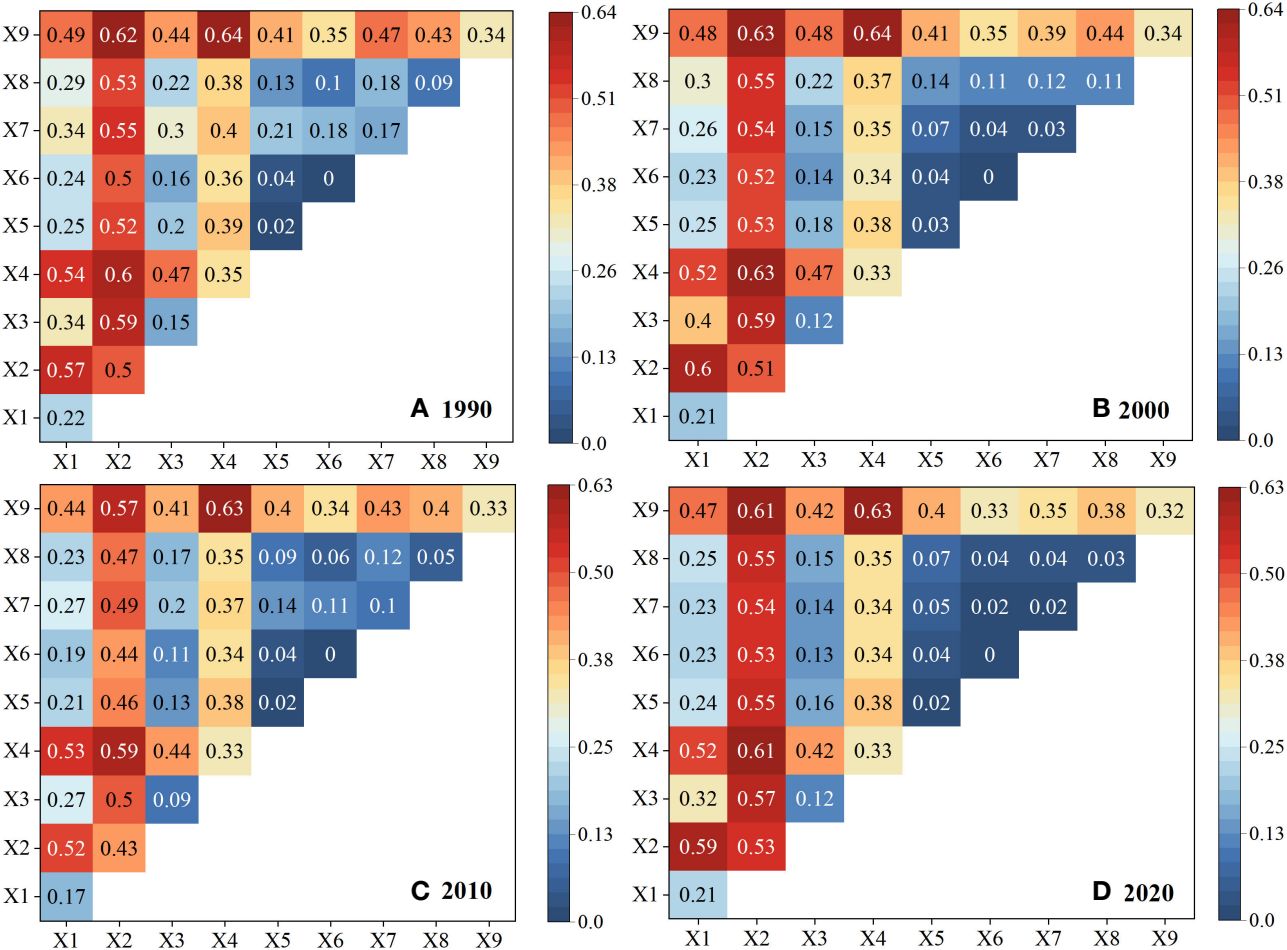
The interactions any two driving factors of theoretical livestock carrying compacity dynamics in 1990,2000,2010 and 2020(X1-X9: Theoretical livestock carrying compacity, Average temperature, Precipitation, scpdsi, Elevation, Slope, Aspect, Population density, GDP, SOM). **(A–D)** correspond to the years 1990, 2000, 2010, and 2020 respectively.

The interaction detector is utilized to identify the interaction effect between two influencing factors, whether the combined effect of the influencing factors Xa and X_b_ increases or decreases the explanatory power for the Y value. As shown in **Figure 10**. Theoretical carrying capacity remains relatively stable during interannual variations when influenced by the interaction of different factors in four different years. Contrasting with the individual factor’s impact q-value, in any given year, the interaction effects between any two factors are more significant in influencing the theoretical grassland carrying capacity compared to the impact of a single driving factor. Among them, the interaction between annual precipitation, altitude, soil, and other driving factors mainly exhibits a bivariate enhancement, While the interaction between slope, aspect, and other driving factors exhibits a non-linear enhancement. In the interplay of theoretical carrying capacity, the strongest interactions are observed between annual precipitation and altitude, annual precipitation and soil organic matter, altitude and soil organic matter, and annual precipitation and annual average temperature. Once again, it is reaffirmed that annual precipitation, altitude, and soil organic matter are the primary driving factors of spatial-temporal dynamics of livestock carrying capacity in Xinjiang grassland. Furthermore, the individual impacts of scpdsi drought index, slope, aspect, and GDP are relatively weak, but soil organic matter significantly enhances their interactive effects on the theoretical carrying capacity of grassland. The interactions among these factors may have affected vegetation growth, soil fertility, and water retention capacity through precipitation. As the altitude increases, the decomposition rate of soil organic matter may be influenced, and high-altitude regions are more sensitive to changes in precipitation, further impacting the carrying capacity of grasslands. The individual impacts of the scpdsi drought index, slope, aspect, and GDP are relatively weak, but soil organic matter significantly enhances their interactive effects on the theoretical carrying capacity of grasslands. This suggests that soil organic matter may indirectly affect the influence of other socio-economic factors on grassland carrying capacity by improving soil moisture conditions and increasing vegetation cover, thereby enhancing their overall impact. Population growth leads to overgrazing of grasslands, and land use changes resulting from economic development can exacerbate land degradation, reduce soil water and nutrient retention capacity, and decrease vegetation cover. In conclusion, these findings further emphasize the complexity of the combined impacts of natural and socio-economic factors on grassland carrying capacity.

## 4 Discussion

### 4.1 Model accuracy validation

In this study, the CASA model is used to simulate the aboveground net primary productivity (NPP) in Xinjiang from 1982 to 2020. This simulation is based on preliminary data such as solar radiation and NDVI (Normalized Difference Vegetation Index) data, which are required inputs for the CASA model. The resulting NPP data are then used as preliminary data for calculating the theoretical carrying capacity of livestock in Xinjiang in this study. After comparing and validating the actual measurements from 2018 to 2020, it was found that the accuracy of this study meets the research requirements, with an R^2^ value of 0.746 (P<0.01) (**Figure 1**). This indicates a satisfactory level of precision in the model and allows for further research calculations. Currently, there have been significant advancements made by numerous scholars in simulating and estimating NPP (Net Primary Productivity). In this study, the average NPP simulation from 1982 to 2020 was calculated as 118.9gCm^-2^. Zhang and Fang X used the CASA model and an improved model to simulate the average NPP in Xinjiang for the periods 2000-2014 and 1982-2013, respectively, resulting in average NPP values of 113.5gCm^-2^ (Zhang et al., 2021)and 155.9 ± 2.74gCm^-2^ (Fang et al., 2019). Comparisons with existing studies reveal that there are variations in the accuracy of NPP simulations to some extent due to factors such as the quality of input data, model parameters, and study periods (Liu et al., 2023b).

In order to accurately calculate the carrying capacity of grasslands in Xinjiang, this study strictly adhered to national standards and assigned specific parameters to the variables in the empirical model. Theoretical stocking rates for Xinjiang from 1982 to 2020 were simulated using the empirical model algorithm at monthly, seasonal, and yearly scales. In previous studies, a common approach was to use NDVI (Normalized Difference Vegetation Index) along with field measured data to employ empirical models or machine learning techniques for estimating aboveground biomass and subsequently inferring theoretical stocking rates (Li et al., 2014; Yu et al., 2021; Wu et al., 2023a). Indeed, This method is more suitable for small-scale studies. When dealing with complex grassland types and regions with significant spatial variations like Xinjiang, achieving high-precision simulation while ensuring accuracy would indeed require extensive collection of field measurements, with uniformly distributed sampling points. This can be a resource-intensive task, requiring significant human and financial resources. Therefore, in this study, based on the extension of the time series, we combine the calculation method used in previous research and utilize an empirical formula that incorporates monthly net primary productivity (NPP) and monthly average temperature to estimate aboveground biomass. This enables us to achieve a finer-scale inversion of theoretical livestock carrying capacity, specifically at monthly and seasonal levels. The theoretical livestock carrying capacity results are in line with the phenological characteristics of Xinjiang. The highest theoretical carrying capacity is observed during the months of June to August, indicating that the higher grass production during the summer season has an impact on the theoretical carrying capacity. The calculation of theoretical livestock carrying capacity typically involves considering the productivity of the grassland and the growth rate of plants. Climate, precipitation, and the rate of plant growth are closely related to the calculation of theoretical carrying capacity and the management of livestock feeding. These factors influence the available forage and the ability of the grassland to support livestock. The higher the productivity and growth rate of plants, coupled with favorable climatic conditions and sufficient precipitation, the higher the potential carrying capacity for livestock. It is important to consider these factors in order to effectively manage livestock and ensure sustainable grazing practices. When grassland productivity increases, theoretically it can support more livestock as there is a greater food supply available. However, it is still important to consider the sustainable utilization of grassland to prevent overgrazing and adverse impacts on the ecological environment. The climate conditions in autumn and winter can lead to a decreased growth rate in grassland vegetation, resulting in a lower forage yield. This, in turn, affects the carrying capacity of livestock, and they may require additional supplementary feed to meet their energy and nutritional needs, exacerbating the shortage of fodder (Sun et al., 2020). Fetzal also indicates that seasonal constraints play a more significant role in determining the intensity of livestock grazing on natural grasslands compared to the annual variation in carrying capacity (Fetzel et al., 2017). The findings of this study once again validate this conclusion and provide further evidence of its reliability within the Xinjiang region.

### 4.2 Temporal and spatial characteristics of grass-livestock balance

Our study analyzed the dynamic changes in the theoretical carrying capacity of livestock in Xinjiang over the past 39 years. We compared these data with the actual livestock numbers from the statistical yearbooks at the county level to assess the grass-livestock balance in each county and examine its interannual variations over the past 33 years. Our findings show that, overall, the grass-livestock balance has been deteriorating and experiencing overgrazing trends over the past 33 years. However, there has been a positive improvement from 2010 to 2016, mainly attributed to the implementation of various policies such as grassland conservation and grazing bans. Based on the analysis of the theoretical carrying capacity of livestock from 1982 to 2020, our predictions suggest a potential reversal of the increasing trend over the past 39 years. This indicates a decrease rather than an increase in the theoretical carrying capacity of grasslands in the future. It suggests that the aboveground biomass of grassland is undergoing degradation under the influence of multiple environmental factors, and the available biomass may not be sufficient to meet the forage needs of grazing livestock. Ren argues that the steady growth in the demand for beef and mutton has resulted in a rise in the population of livestock over the past few decades (Ren et al., 2021). This has resulted in overgrazing and a significant reduction in the availability of grassland resources in many grassland areas. Indeed, overgrazing has a significant impact on grasslands. It results in excessive consumption of grassland vegetation, hindering its ability to regenerate fully and leading to grassland degradation. Frequent overtrampling and overgrazing by livestock can damage the growth points of plants and the topsoil, thereby reducing grassland coverage and vegetation diversity (Zumo et al., 2021). Within sensitive areas, even minimal grazing can have a substantial impact on the abundance or diversity of species within a community (Filazzola et al., 2020). However, in low-pressure ecosystems, grazing can stimulate plant growth through compensatory responses, thereby reducing the impact of livestock on plant communities (Ramula et al., 2019). Properly managed grazing not only helps stimulate compensatory growth in plants and accelerate nutrient cycling but also influences the physical structure of the soil, promoting root development and thus indirectly impacting grassland productivity and plant diversity (Wei et al., 2022).

To effectively alleviate and possibly eliminate the current overgrazing situation in Xinjiang grasslands, it is necessary to rely on a combination of various measures and policies for effective management and control (WANG Lijing et al., 2022). From 46 counties in 1988 to 58 counties in 2020 (**Figure 8B**), the number of counties experiencing different levels of overgrazing has gradually increased. Moreover, the degree of overgrazing in severely affected counties far exceeds the carrying capacity of the local grassland resources. It is crucial to address this issue through effective measures and policies to restore the balance and sustainability of the grassland ecosystem in those regions. Therefore, it is necessary to implement graded management for counties and cities experiencing different levels of overgrazing, optimize the layout of the livestock industry, and prioritize the control of counties and cities with persistent moderate to severe overgrazing. This is to prevent the overgrazing situation from further deteriorating. In addition, counties experiencing mild levels of overgrazing should be subjected to policy-based management. This can include measures such as regular rotational grazing, reducing the grazing time, strictly controlling the grazing density, or implementing grassland improvement practices. On the other hand, counties with grassland ecosystems that are not overgrazed, i.e., below the threshold, should be maintained within the “safe ecological limits” to ensure their ecological stability and sustainability (Li et al., 2023a).

### 4.3 The driving factors of theoretical carrying capacity of livestock

Based on the above results, it was found that the impact of the nine driving factors on grassland carrying capacity was not consistent during different periods (1988, 2000, 2010, and 2019). This could be due to various reasons. Climate factors such as annual precipitation and average annual temperature may have changed over time, leading to different degrees of impact on grassland carrying capacity. Geographic factors like altitude, slope, and aspect may vary across regions, and different geographical conditions may be related to the structure and function of grassland ecosystems, thereby affecting carrying capacity. Human factors such as population density and Gross Domestic Product (GDP) may have experienced different disturbances and pressures in different periods. For example, population growth results in overgrazing of grasslands, and land use changes due to economic development can also impact carrying capacity. In conclusion, grassland carrying capacity is influenced by multiple driving factors, including climate factors, geographic conditions, and human activities. The variations and interactions of these factors may lead to inconsistent changes in grassland carrying capacity over time, and further highlight the combined impacts of natural and human activities on grassland carrying capacity.

Research suggests that human activities and climate change are causing desertification or degradation in grasslands located in arid and semi-arid regions (Zhou et al., 2023). Xinjiang is situated in the northwestern part of China, with low annual rainfall in the region. The effects of climate change, including shifts in yearly precipitation patterns and more frequent drought occurrences, poses a significant vulnerability to the vegetation found in alpine grasslands and desert grasslands. This study indicates that over the past 39 years, the livestock carrying capacity in Xinjiang has been fluctuating and closely correlated with factors such as annual rainfall, altitude, and soil conditions (Cheng et al., 2017). This corresponds with Umuhoza’s research findings (Umuhoza et al., 2021), which state that climate, soil types, topography plays a crucial role in shaping the composition and projected distribution of grassland carrying capacity. Luo also pointed out that the degradation of grassland could be caused by factors like small mammals, climate change, challenging environments, weak soil, and over-grazing (Luo et al., 2021). Indeed, factors such as reduced precipitation, increased interannual variability in precipitation, and climate warming can potentially lead to a significant decrease in grassland productivity and livestock carrying capacity (Li et al., 2018). Global climate warming has led to increased evapotranspiration, decreased precipitation, and reduced grassland coverage, posing a serious risk of degradation to grasslands in Xinjiang (Majid, 2023). Grassland degradation not only causes significant damage to the ecological environment but also has a great impact on animal husbandry. The decrease in grassland coverage and forage yield leads to a reduction in carrying capacity for livestock. This decrease in available grazing resources results in a decline in the carrying capacity of grasslands. Climate change has a substantial influence on the species composition, growth rate, and process of species accumulation in grassland ecosystems. Therefore, temperature and precipitation are essential key climate factors in the growth and accumulation processes of grassland biomass, and their accuracy is crucial for studying biomass accumulation in grasslands (Song et al., 2022). The organic carbon content in the soil of different grassland types generally follows a consistent trend with soil depth. As the soil depth increases, there is a decrease in soil porosity, an increase in soil bulk density, and a weakening of the decomposition capacity of soil microorganisms in the lower layers. In the long run, The increase in soil organic carbon storage can have a positive feedback on plant productivity (Zhou et al., 2023). The escalating contradiction between the limitless growth of population and the finite nature of resources has intensified the negative feedback of human activities such as overgrazing, grassland cultivation, and mineral exploitation on the sustainability of grassland ecosystems (Fang et al., 2021). China has made significant progress in enhancing grassland carrying capacity since 2000 through the implementation of various projects focused on grassland conservation and ecological restoration. This highlights the positive impact of human efforts in this area. Major conservation projects, including the Conversion of Cropland to Forest Program, the Grazing Ban and Grassland Restoration Project, along with grassland ecological conservation incentive policies, have made remarkable efforts towards the rehabilitation of grassland vegetation in China. These endeavors have been instrumental in enhancing the overall health and endurance of grassland ecosystems. These projects have greatly improved the carrying capacity of grasslands and enabled precise management and control (Ge et al., 2022).

### 4.4 Uncertainties and future research

We have explored the estimation of grassland aboveground biomass based on NPP, further calculating the theoretical carrying capacity, assessing the grass-livestock balance in Xinjiang grasslands, and quantitatively examining the influencing factors. However, there are still uncertainties that need to be addressed. First of all, the NDVI data used in this study may be affected by factors such as cloud cover, solar angle, and atmospheric conditions (Ma et al., 2022). Therefore, when estimating aboveground biomass and theoretical carrying capacity of grassland based on the input data, the relatively low spatial resolution of vegetation index data may not accurately reflect the actual condition of vegetation within the 1km x 1km area, and there may also be some inaccuracies in the grassland distribution data (Shen et al., 2021). Secondly, the calculation of aboveground biomass only considered the inclusion of average temperature to simulate theoretical carrying capacity. However, after conducting an analysis of influencing factors, it was found that factors such as precipitation, soil organic matter, and altitude had much higher correlations than average temperature. Moreover, factors such as grassland vegetation coverage and density also have significant relationships. In our next study, we plan to validate our research results by conducting multivariate modeling of closely related factors (such as altitude, precipitation, etc.) to better simulate aboveground biomass, optimize model parameters, and achieve more precise simulation models. Lastly, in calculating the theoretical carrying capacity, we only considered the utilization rate of different grassland types, overlooking factors such as rodent and insect damage, which can lead to grassland degradation. In future research, we plan to comprehensively consider factors such as disease and pest loss rates, distance from water sources, altitude, slope, etc., and incorporate them into the calculation model for livestock carrying capacity. Therefore, in future research, it is necessary to comprehensively integrate the above-mentioned factors and research methods for model indicator selection and model parameter optimization, in order to gain a more comprehensive understanding of the balancing effects between grassland and livestock development.

## 5 Conclusion

This study utilized long-term remote sensing data from 1982 to 2020 to estimate the Net Primary Productivity (NPP) and simulate aboveground biomass in grasslands in Xinjiang, China. The theoretical carrying capacity was then calculated, and a spatiotemporal analysis was conducted at monthly, seasonal, and annual scales, as well as for different grassland types. By integrating statistical yearbook data, the study explored the grass-livestock balance and its interannual variation trends at the county level in Xinjiang. The research findings offer scientific guidance and decision-making support for the region’s coordinated and sustainable development of grassland resources and the livestock industry. The conclusions are as follows:

(1) The potential number of livestock that could be sustained showed an upward trend in Xinjiang from 1982 to 2020 and has since stabilized. The spatial distribution indicates a gradual decrease from north to south and from east to west. The average value is 7.48 SU/hm^2^. At the seasonal scale, the theoretical carrying capacity of livestock in Xinjiang follows the order from highest to lowest: growing season > summer > spring > autumn > winter. At the monthly scale, the month of July exhibits the highest carrying capacity for livestock. In terms of different grassland types, the annual theoretical carrying capacity of livestock per unit area in Xinjiang follows the order from highest to lowest: meadow > alpine subalpine meadow > plain grassland > desert grassland > alpine subalpine grassland. However, it is worth noting that in the future, the theoretical carrying capacity of grasslands for livestock may show a decreasing trend, which could be opposite to the past trend.
(2) From 1988 to 2020, the average grass-livestock balance index in Xinjiang was 2.61%, indicating a mild state of overgrazing. Overall, there was an upward trend in the balance index during this period.At the county level, compared to the year 1988, the number of counties experiencing overgrazing increased from 46 to 59 and then to 65 in the years 2000 and 2010 respectively. However, by the year 2020, there was a slight decrease in the number of overgrazed counties, dropping from 65 to 58 compared to the previous two years.
(3) According to single-factor analysis of the geographical detectors, the q-value ranking from highest to lowest impact on grassland carrying capacity in Xinjiang is: annual precipitation > elevation > soil organic matter > mean annual temperature > ScPDSI drought index > population density > GDP > slope > aspect. Furthermore, the factor interaction detector confirms that annual precipitation, elevation, and soil organic matter are the main driving factors for the spatiotemporal dynamics of grassland carrying capacity in Xinjiang. The individual influences of SCPDSI drought index, slope, aspect, and GDP are relatively weak. However, soil organic matter significantly enhances their interaction effects on the theoretical carrying capacity of grasslands.

## Data availability statement

The raw data supporting the conclusions of this article will be made available by the authors, without undue reservation.

## Author contributions

LM: Data curation, Formal analysis, Investigation, Methodology, Resources, Visualization, Writing – original draft. JZ: Conceptualization, Project administration, Supervision, Validation, Writing – review & editing. JP: Funding acquisition, Investigation, Resources, Software, Validation, Writing – review & editing. XX: Funding acquisition, Investigation, Project administration, Resources, Validation, Writing – review & editing. YL: Conceptualization, Data curation, Methodology, Resources, Supervision, Writing – review & editing. LL: Conceptualization, Formal analysis, Investigation, Methodology, Supervision, Writing – review & editing. WH: Conceptualization, Formal analysis, Supervision, Visualization, Writing – review & editing. GL: Funding acquisition, Investigation, Project administration, Resources, Validation, Writing – review & editing. JZ: Funding acquisition, Investigation, Project administration, Resources, Validation, Writing – review & editing.
